# Genome-wide analysis of the U-box E3 ubiquitin ligase family role in drought tolerance in sesame (*Sesamum indicum* L.)

**DOI:** 10.3389/fpls.2023.1261238

**Published:** 2023-09-19

**Authors:** Hengchun Cao, Qiuzhen Tian, Ming Ju, Yinghui Duan, Guiting Li, Qin Ma, Haiyang Zhang, Xianmei Zhang, Hongmei Miao

**Affiliations:** ^1^ Henan Sesame Research Center, Henan Academy of Agricultural Sciences, Zhengzhou, Henan, China; ^2^ The Shennong Laboratory, Zhengzhou, Henan, China; ^3^ Key Laboratory of Specific Oilseed Crops Genomics of Henan Province, Henan Sesame Research Center, Henan Academy of Agricultural Sciences, Zhengzhou, Henan, China; ^4^ Luohe Academy of Agricultural Sciences, Luohe, Henan, China

**Keywords:** ubiquitin, *Sesamum indicum*, PUB, drought tolerance, gene family

## Abstract

Plant U-box (PUB) proteins belong to a class of ubiquitin ligases essential in various biological processes. Sesame (*Sesamum indicum* L.) is an important and worldwide cultivated oilseed crop. However few studies have been conducted to explore the role of *PUB*s in drought tolerance in sesame. This study identified a total of 56 members of the sesame *PUB* family (*SiPUB*) genes distributed unevenly across all 13 chromosomes. Based on phylogenetic analysis, all 56 *SiPUB* genes were classified into six groups with various structures and motifs. *Cis*-acting element analysis suggested that the *SiPUB* genes are involved in response to various stresses including drought. Based on RNA-seq analysis and quantitative real-time PCR, we identified nine *SiPUB* genes with significantly different expression profiles under drought stress. The expression patterns of six *SiPUB* genes in root, leaf and stem tissues corroborated the reliability of the RNA-seq datasets. These findings underscore the importance of *SiPUB* genes in enhancing drought tolerance in sesame plants. Our study provides novel insights into the evolutionary patterns and variations of *PUB* genes in sesame and lays the foundation for comprehending the functional characteristics of *SiPUB* genes under drought-induced stress conditions.

## Introduction

With the escalation of global climate change, the impact of the environment on crop production is becoming increasingly severe (Vicente-Serrano et al., 2020). Drought is recognized as a significant environmental stressor, and its impact on crop production surpasses the cumulative yield losses caused by all pathogens combined (Gupta et al., 2020; Hoover et al., 2021). Drought stresses can occur at any stage of plant growth, leading to water deficit and harsh reactions. Many signaling pathways in plants are activated under drought stress, regulating the expression of different genes and generating multiple defense proteins and protective molecules. For example, abscisic acid (ABA), an essential component of plant response to abiotic stresses, including drought, activates various genes through SnPK2, triggering stomatal closure and improving water balance (Cheng et al., 2017; Lin Z. et al., 2021). In *Arabidopsis thaliana*, *BRL3* allows plant growth and survival under drought stress by promoting the accumulation of osmoprotectant metabolites in root tissues (Fàbregas et al., 2018). Therefore, it is necessary to understand the molecular mechanism of plant responses to drought stress and develop drought-resistant crops.

The Ubiquitin-Proteasome System (UPS) is a major pathway that regulates the post-translational level of proteins and is essential in plant development and plant-environment interactions (Bing et al., 2016). The classic UPS contains several members, including ubiquitin (Ub), Ub-activating enzyme (E1), Ub-binding enzyme (E2), Ub ligase (E3), deubiquitinating enzyme (DUB), and 26S proteasome (Bhattacharyya et al., 2014). E3 can specifically recognize the ubiquitin target proteins, which are crucial in the ubiquitin pathway (Hu and Hochstrasser, 2016). E3 can be classified into four categories according to their functional domains: RING, HECT, U-box, and cullin. The U-box domain is conserved in nearly all eukaryotes with a length of ~70 amino acids. Plant U-box (PUB) proteins are central to many biological processes, such as seed germination, hormonal response, and biotic and abiotic stresses (Hu and Hochstrasser, 2016). Numerous crops involve the diverse functions of *PUB*s in response to various abiotic stresses, including cold, salinity, high temperature, and drought (Trujillo, 2018; Cui et al., 2023). Under normal conditions, E3 can inhibit the drought stress signaling pathways, thus eliminating negative regulators that respond to stimulation or reduction of the stress signaling pathway (Al-Saharin et al., 2022). In addition, E3 may act as positive feedback factors to enhance signaling under drought stresses and eliminate stress-induced signaling pathways when conditions return to normal, allowing the plant grow further (Liu et al., 2021).

Many studies have demonstrated the significant impact of *PUB*s on numerous crops subjected to drought stress. For example, by degrading leucine-rich repeat protein 1 (LRR1) and kinase 7 (KIN7), *AtPUB11* negatively regulated drought tolerance (Chen et al., 2021). *AtPUB22* and *AtPUB23* are negative regulators of drought tolerance in *A. thaliana*. The functional loss of these two genes could significantly improve drought tolerance, while their overexpression would decrease it. Further experiments indicated that *AtPUB22* and *AtPUB23* could synergistically control drought signaling pathways, mainly because their double mutants displayed greater tolerance (Cho et al., 2008). In poplar, *PalPUB79* might positively regulate ABA-dependent drought tolerance through the ubiquitination of *PalWRKY77*. The overexpression of *PalPUB79* enhances drought tolerance, while the *PalPUB79* RNAi lines display more sensitivity (Tong et al., 2021). The hot pepper *CaPUB1* exerts a negative regulatory effect on drought responses in both *A. thalina* and *Oryza sativa*. The overexpression of *CaPUB1* leads to the development of drought-sensitive phenotypes in plants (Cho et al., 2006; Hye Jo et al., 2016). In addition, *OsPUB41* acts as a crucial negative regulator in drought tolerance in rice (Seo et al., 2021).

Sesame (*Sesamum indicum* L.) is one of the oldest oilseed crops within the genus *Sesamum* of the *Pedaliaceae* family (Ashri, 1998; Zhang et al., 2013). Due to its high oil (~58%) and protein content (~25%) and its excellent antioxidant and acidic properties, the sesame seed is commonly referred to as the “oilseed queen” (Bedigian and Harlan, 1986; Zhang et al., 2019). Compared to other oilseed crops, such as soybean (*Glycine max*), sunflower (*Helianthus annuus* L.), and oilseed rape (*Brassica napus*), sesame has higher oil content and tolerance to drought and high temperatures and can display high yields in tropical and subtropical climates (Zhang et al., 2013; Zhang et al., 2019). Even though sesame can survive in semi-arid conditions, its nutrition quality and seed yield can be affected by drought stress at the vegetative and reproductive growth stages (You et al., 2019). To our knowledge, the key role of the *PUB* gene family in plantlet growth and stress response has not been fully identified in sesame. In this study, we conducted a systematic characterization of *SiPUB* genes and a detailed analysis of their evolution and structures. Based on an RNA-seq dataset, we investigated the expression profiles of *SiPUBs* under drought stress and confirmed their differential expression by quantitative real-time PCR (qRT-PCR). Our study provides valuable information for further studying the evolution and function of *PUB* genes in sesame.

## Material and methods

### Genome identification of U-box gene family members in sesame

To identify potential members of the *PUB* gene family, this study utilized our latest sequenced sesame genome data (GenBank under the accession MBSK00000000 in BioProject no. PRJNA315784) (Miao et al., 2021). The seed file of the U-box domain (PF04564) was obtained from the Protein Families Database (Pfam) v35.0 (Finn et al., 2014). HMMER (v3.3.2) was used to search the U-box domain in the sesame protein sequences (Potter et al., 2018). Additionally, we performed homologous searching using BLAST+ (v2.8.1) based on the *PUB* genes in *Arabidopsis* (Camacho et al., 2009). Finally, we used PfamScan (v1.6) to confirm the presence of the U-box domain in the candidate genes (Li et al., 2015). Only *SiPUB* genes with a complete the U-box conserved domain could be considered for further analysis.

### Phylogenetic analysis of SiPUB proteins

MAFFT (v7.505) was employed to perform multiple sequence alignment in this study (Katoh and Standley, 2013). The phylogenetic tree was constructed by the FastTree v2.1.11 with a bootstrap of 1000 replications using the recommended models (Price et al., 2010). The software FigTree (v1.4.4) was used to display the phylogenetic tree of SiPUB proteins (http://tree.bio.ed.ac.uk/software/Figtree/). Sequence alignments were visualized and displayed using the online LaTeX editor Overleaf (https://www.overleaf.com/). To explore the diversity of *SiPUB*s, we obtained the amino acid sequences of PUBs in rice and *A. thaliana* from previous studies (Wiborg et al., 2008; Zeng et al., 2008).

### Structural characteristics, motif and cis-acting elements analysis

To identify the conserved motifs of *SiPUB*s, we utilized the Multiple Expectation Maximization for Motif Elicitation (MEME) Suite web server with parameters set to maximum motifs of 10 (Bailey et al., 2009). TBtools was applied to perform the intron-exon gene structures with the annotation files of sesame (Chen et al., 2020). The 2,000bp upstream sequence of *SiPUB* genes was extracted as the putative promoter sequence, and then utilized in the PlantCare database to identify *cis*-acting elements with default parameters (Lescot et al., 2002). The sequence length, molecular weight (MW), and isoelectric point (pI) were obtained on the ExPasy website (Duvaud et al., 2021).

### Chromosomal and subcellular location, synteny analysis

The software of TBtools was used for pairwise comparisons of the genomes of sesame, rice, and *A. thaliana* (Chen et al., 2020). The online tool WoLF PSORT was employed to predict the subcellular localization of SiPUB proteins (Horton et al., 2007).The synteny regions and chromosome distribution were performed by MCScanX with default parameters and then used Circos to visualize these results (Krzywinski et al., 2009; Wang et al., 2012). The chromosome location map was generated using MG2C v2.1 (http://mg2c.iask.in/mg2c_v2.1/), based on the positions of *SiPUB*s (Chao et al., 2021).

### RNA-Seq data analysis

To analyze the expression levels under drought stress, we obtained the Fragments Per Kilobase of exon model per Million mapped fragments (FPKM) matrix data of drought-tolerant genotype TEX-1 and drought-sensitive genotype VEN-1 from the NCBI database (accession number GSE148340) (Song et al., 2021). The log2 fold change (log2FC) of each treatment was calculated and applied to the R package “pheatmap” to generate the expressed heatmap.

### The three-dimensional structure and PPI analysis of SiPUB proteins

To investigate the 3D structures of SiPUBs, we employed the RCSB Protein Data Bank (RCSB PDB) through comparative modeling (Rose et al., 2017). PyMOL (v2.5.4) was used to visualize and analyze 3D structures of the proteins (Yuan et al., 2017). Furthermore, the online resource Search Tool for the Retrieval of Interacting Genes (STRING) was used to predict protein-protein interactions (PPIs) with an interaction score of 0.4 or higher (Szklarczyk et al., 2021). This threshold ensures a high level of confidence in the connections between nodes and lines. Cytoscape (v3.9.1) software was utilized for visualization and analysis of the PPI networks (Saito et al., 2012).

### Plant materials and drought stress treatments

The sesame seeds of drought-sensitive YM1 (Henan No. 1) and YM 5 (Yuzhi No. 3), and drought-tolerant YM19 (Zhengheizhi No.1) and YM20 (Zhengheizhi No.4) were collected from our experimental field (the sesame germplasm orchard of Henan Sesame Research Center at Yuanyang in Henan province). Then, the sesame seeds were surface-sterilized in 3% sodium hypochlorite solution for 7 min, followed by four times washing with sterile water. Twelve seeds were sown in each plot with an equal weight of sterilized vermiculite, and ten seedlings were selected per pot during the stage when the first pair of true leaves emerged. The seedlings were cultivated in an HP1500GS-B type artificial climate incubator. The cultivation conditions were set as follows: a photoperiod of 15h light and 9h darkness, temperature at 28°C, relative humidity of 70%, and a light intensity of 20,000 lux. Both the control group and the treatment group were watered with a quantitative nutrient solution in each pot. The seedlings were cultivated until the stage of two pairs of true leaves. The treatment group was subjected to drought stress treatment without watering, while the control group was maintained regular watering conditions. Samples from both the control and treatment groups were collected at 8, 9, 10, and 12 days.

### RNA isolation and quantitative RT-PCR analysis

The RNA extraction from three tissues (root, stem, and leaves) of control and treatment was performed using the Plant Total RNA Isolation Kit Plus (FOREGENE, China). Subsequently, the PrimeScript™ RT reagent kit (Takara Bio, China) was utilized to reverse transcribe the extracted RNA into cDNA. For qRT-PCR analysis, the Roche LightCycler® 480 II instrument (Roche, Mannheim, Germany) was employed with LightCycler® SYBR GREEN I Master Mix kit (Roche, China). We designed 7 pairs of specific primers (**Supplementary Table S1**) using Primer6.0 software. The relative expression levels of *SiPUB*s were estimated using the 2^−ΔΔCT^ method, with the sesame β-tubulin gene (Sindi_2728600) serving as the internal reference. These analyses were carried out in three independent biological replicates and three technical replicates of each biological replicate.

## Results

### Genome-wide identification of *PUB* genes in sesame

In this study, 56 *PUB* genes with their complete U-box domain were obtained (**Supplementary Table S2**). These *PUB* genes were renamed *SiPUB1* to *SiPUB56* based on their location orders on the 13 sesame chromosomes. To characterize these *SiPUB* genes, we analyzed the length of their open reading frame (ORF) and the expected protein sequence, molecular weight (MW), isoelectric points (pI), and subcellular and chromosomal location (**Supplementary Table S2**). The CDS length of *SiPUB* genes varied from 843bp (*SiPUB12*) to 4,500bp (*SiPUB52*), with an average of 1,817bp. SiPUB52 is the longest U-box protein in sesame and comprises 1,499 amino acids, whereas SiPUB12 is the shortest, with 280 amino acids (**Supplementary Table S2**). MW of the 56 proteins ranged from 32.20 to 167.04 kDa, with an average of 67.06 kDa. The pI value ranged from 5.21 (SiPUB28) to 8.91 (SiPUB18). Based on their pI, 30 PUBs were classified as acidic proteins, and 26 as basic proteins. Additionally, 42 SiPUBs were grouped as hydrophilic (GRAVY <0) and 14 as hydrophobic (GRAVY>0). The most hydrophilic protein was SiPUB43, and the most hydrophobic was SiPUB6. The subcellular location of *SiPUB*s was predicted by WoLF PSORT, indicating that most PUB proteins were located on the nuclear (20), cytoplasm (16), chloroplast (12), and plasma membrane (4), except three located in the mitochondrion and one located in the endoplasmic reticulum (**Supplementary Table S2**).

### Phylogenetic relationship of *PUB* genes

**Figure 1:**
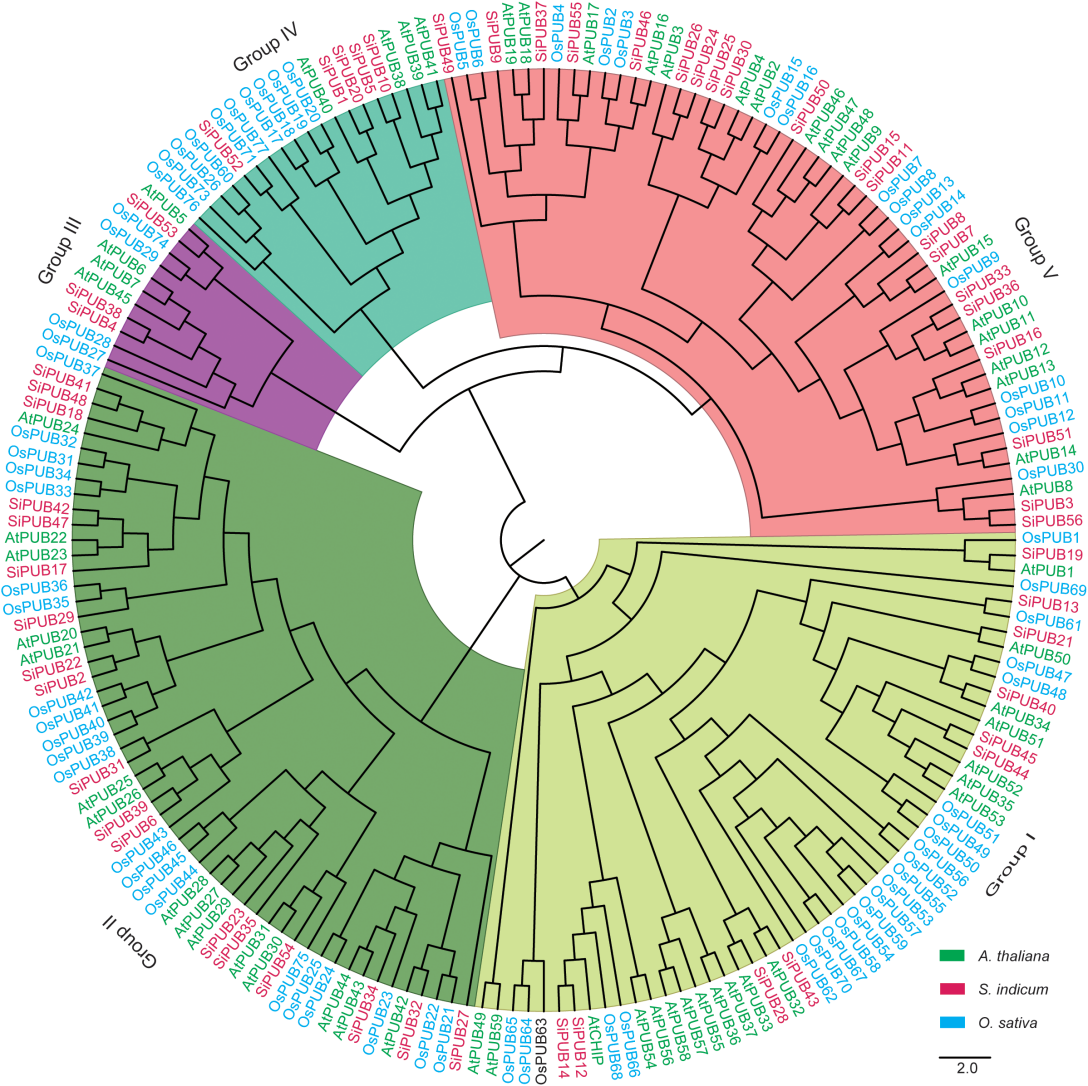
Phylogenetic tree of *PUB* gene family. The phylogenetic tree was constructed using the maximum likelihood (ML) method with 1,000 bootstraps. All of 192 PUB proteins are clustered into five distinct subgroups, namely Group I-V clades in different colors. U-box proteins in *A. thaliana*, *S. indicum*, and *O. sativa* are labeled in green, pink, and blue, respectively.

To verify the various evolution characters of the *PUB* gene family, 192 PUB proteins (56 from sesame, 76 from rice, and 60 from *A. thaliana*) (**Supplementary Table S3**) were used to construct a phylogenetic tree (**Figure 1**). The phylogenetic analysis indicated that these PUB proteins were divided into five subgroups. Ten *SiPUBs* belong to the same subgroup as *Arabidopsis* and rice U-box proteins (Group I), with possible similar functions. Group II was the largest, with 55 *PUB* genes, whereas Group III had the smallest number, with 11 *PUB* genes. We found that sesame and *Arabidopsis PUB* genes exhibited a relatively closer relationship compared to rice. For example, Group IV comprised 19 *PUB* genes, among which four sesame (*SiPUB1*, *SiPUB20*, *SiPUB5*, and *SiPUB10*) and four *Arabidopsis* (*AtPUB40*, *AtPUB38*, *AtPUB39*, and *AtPUB41*) genes were clustered together, whereas one sesame *PUB* gene (*SiPUB52*) was clustered with ten rice *PUB* genes (*OsPUB76*, *OsPUB73*, *OsPUB26*, *OsPUB60*, *OsPUB71*, *OsPUB77*, *OsPUB17*, *OsPUB18*, *OsPUB19*, and *OsPUB20*).

**Figure 2:**
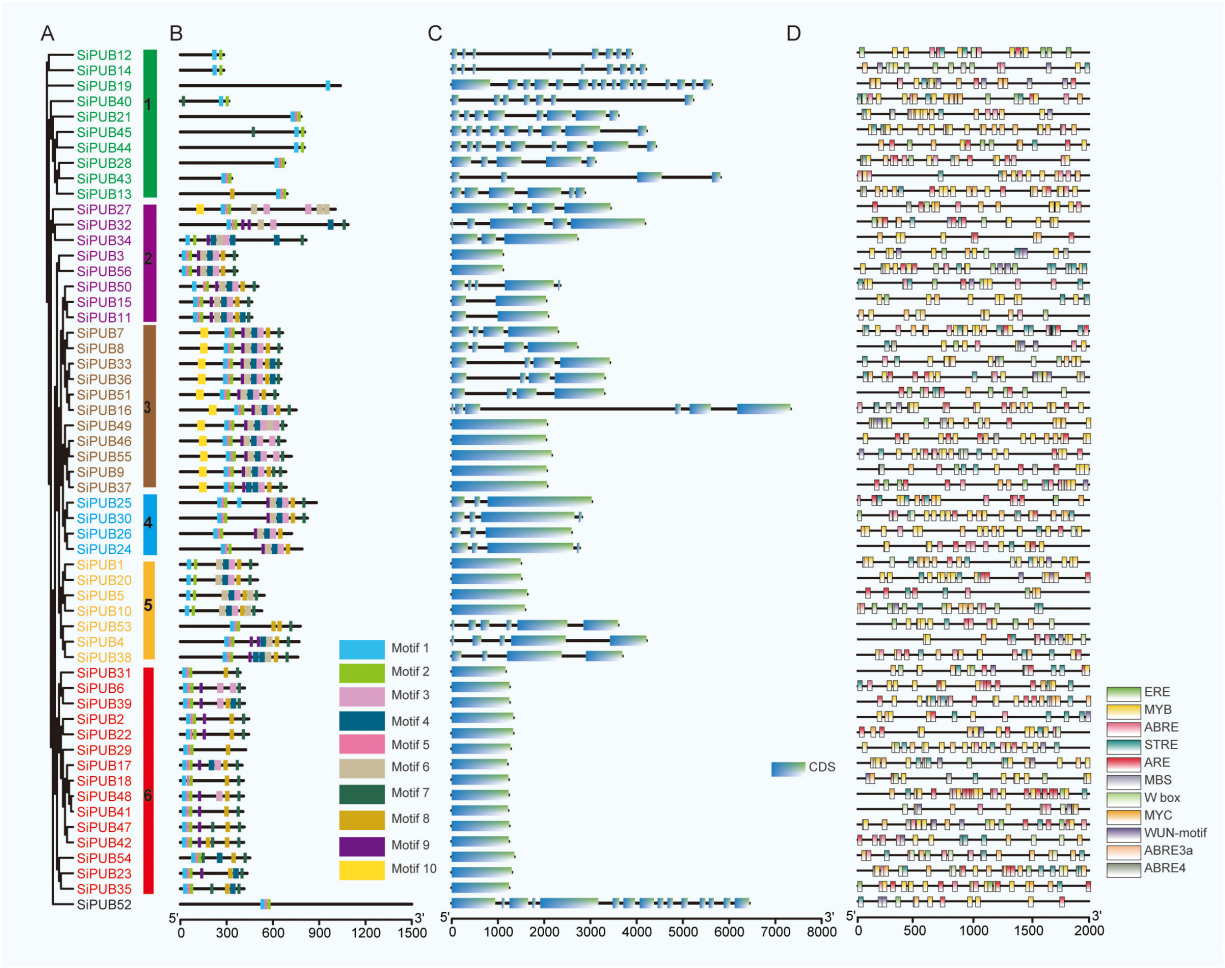
Structural comparison and phylogenetic tree of *SiPUB*s. **(A)** Phylogenetic tree of the 56 *SiPUB*s generated using the maximum likelihood (ML) method. Six distinct groups are shown in different colored squares with gene names. **(B)** Distribution of conserved motifs in SiPUB proteins. Ten different motifs are predicted using MEME software. The scale bar on the right indicates the distance. **(C)** Structure comparison of *SiPUB* genes. Intron and exon are shown in black line and blue rectangle, respectively. **(D)** Distribution of *cis*-acting elements associated with drought stress in the putative promoter of *SiPUB* genes. Eleven elements in the putative promoters of the *SiPUB* gene family are shown. The color scale on the right represents the distance between *cis*-acting elements.

To better understand the phylogenetic relationships of *PUB* genes in sesame, we inferred a phylogenetic tree using FastTree with the Maximum Likelihood (ML) method (Minh et al., 2020). The topology of the phylogenetic tree was divided into six groups (**Figure 2A**), among which Group 6 was the largest with 15 genes, followed by Groups 1 and 3 with 10 and 11 genes respectively. Group 4 was the smallest, with four genes. Among these SiPUB proteins, SiPUB52 displayed an apparent difference from other SiPUBs, and was not clustered into any groups in the phylogenetic tree (**Supplementary Table S2**). The findings are consistent with the phylogenetic analysis of *PUB* genes in the three abovementioned species, as depicted in **Figure 1**, further validating the reliability of the *SiPUB* phylogenetic analysis performed in this study.

### Characterization of *SiPUB* motifs and gene structures

To further investigate the relationships between the 56 SiPUB proteins containing a U-box domain (**Supplementary Table S4**) in the phylogenetic tree, we evaluated the conserved motifs using the program MEME. Ten motifs were identified in 56 *SiPUB* genes, named Motifs 1 to 10 (**Figure 2B**), among which Motifs 1, 2, 5, and 7 were encoded by most *SiPUB* genes and reflected the high motif conservation in SiPUB proteins. Motifs 1 and 2 were determined as conserved U-box sequences necessary to maintain the U-box structure and support the ubiquitin linkage activity. Other domains, such as ARM, PK_Tyr_Ser-Thr, Pkinase, ANAPC3, Ufd2P_core, and WD40, were also identified in sesame U-box proteins. Motif 6 could be a part of the ARM conserved domain, with the U-box-ARM subfamily being the most common type in the U-box family (**Supplementary Table S4**). Furthermore, all the genes in the same phylogenetic group encoded similar conserved motifs, indicating possible similar functions. For example, within Group 3, 11 genes encoded ten distinct motifs (Motifs 1 to 10). These motifs were conserved in quantity and sequential arrangements across the encoded proteins. Many highly conserved amino acids were also detected in the sesame U-box domain (**Supplementary Figure S1**), such as the hydrophobic amino acids of proline (P), phenylalanine (F), and isoleucine (I), the acidic amino acid aspartic acid (D), and the basic amino acid basic amino acid arginine (R), cysteine (C), and serine (S). These amino acids likely have a crucial function in forming ionic or hydrogen bonds and stabilizing the U-box domain.

To understand the composition and function of U-box domains, we analyzed the gene structure of the 56 *SiPUB* genes using conserved sequences and exon/intron positions (**Figure 2C**). The number of exons varied from 1 to 16 in *SiPUBs*, suggesting a complex RNA splicing process. Genes from the same group had similar exon/intron structures. Group 6 *PUB* genes had the fewest exons (1), while Group 1 genes had the most (average 8). The gene structure and conserved motif analysis results were consistent with the *SiPUB* phylogenetic tree, confirming the accuracy of the phylogenetic tree classification.

### Chromosomal localization and gene ontology analysis of *SiPUB* genes

**Figure 3:**
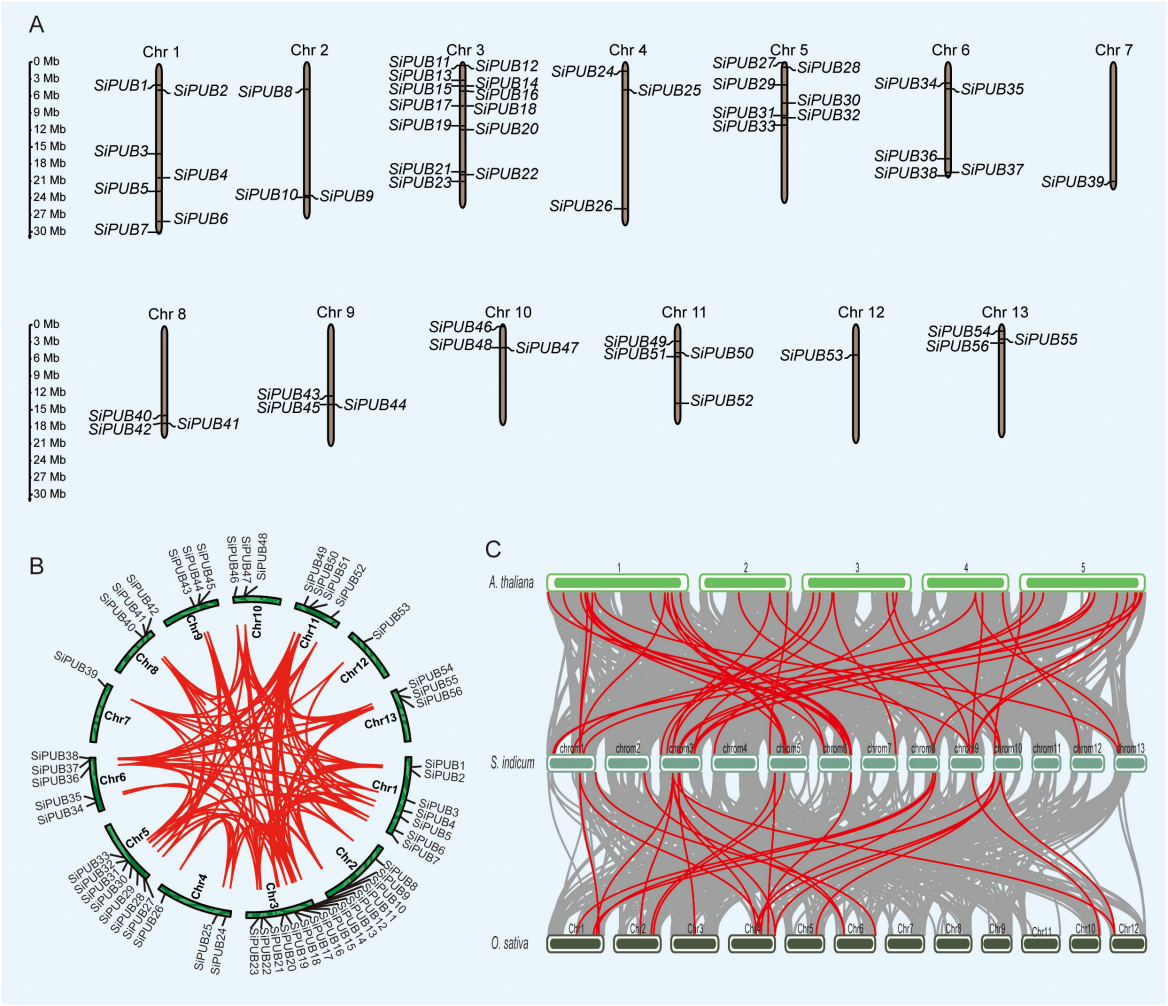
Distribution and synteny results of *SiPUB* genes in sesame genome. **(A)** Distribution and chromosomal localization of *SiPUB* genes. Green columns represent sesame chromosomes. **(B)** The synteny analysis and distribution pattern of *SiPUB* genes. Red lines indicate synteny gene pairs within the *PUB* gene family in sesame. **(C)** Collinearity analysis of the genomes of *A. thaliana*, *S. indicum*, and *O. sativa*. Gray lines represent the overall co-collinearity of all genes between two species. Red lines represent the collinearity specific within the *PUB* gene family.

According to the location in the sesame genome, 56 *PUB* genes were unevenly mapped across 13 chromosomes (**Figure 3A**). *Si*Chr.3 had 13 *PUB* genes, *Si*Chrs.1 and 5 had seven *SiPUBs* each, and only one *PUB* gene was located on *Si*Chrs.7 and 12. The transcription direction of 28 *PUBs* was the same as the sequence provided by the genome, and the other 28 were reversely transcribed. Next, we performed a homolog analysis of *SiPUB* using MCScanX software. Thirty genes were assigned to whole genome duplication (WGD) and segmental duplication, while 23 were derived from dispersed duplication blocks (**Figure 3B**). In addition, three genes were assigned to tandem duplication blocks (**Supplementary Table S5**). No genes were derived from proximal and singleton duplication blocks. Therefore, WGD and segmental duplication events might expand the *PUB* gene family in sesame. Most duplicated genes might be silenced over time, but a few purified selected genes still retain. In the sesame *PUB* gene family, 251 homologous gene pairs were identified, including 56 homologous genes (**Figure 3B**). Furthermore, we detected a collinear relationship between sesame, Arabidopsis, and rice (**Figure 3C**). As a result, 45 orthologous gene pairs were identified between *SiPUBs* and *AtPUBs*, of which 8 *AtPUBs* had two or three orthologous copies in the sesame genome. Meanwhile, 25 orthologous gene pairs were identified between *SiPUBs* and *OsPUBs*, fewer than those between sesame and *Arabidopsis*, suggesting that *SiPUB* genes have a closer relationship with *Arabidopsis PUB*s than rice ones. Identifying these orthologous genes with potential similar functions can promote the study of *SiPUBs* in the future.

### *Cis*-acting elements in the promoter regions of *SiPUB* genes

*Cis*-acting elements play a crucial role in gene regulation. To explore the transcriptional regulation of *SiPUBs*, we predicted the *cis*-acting elements present within the 2,000bp region upstream of the coding sequence using the Plant-CARE database (**Supplementary Table S6**) (**Figure 2D**). Our analysis revealed a total of 8,819 occurrences spanning 95 distinct types of *cis*-acting elements within the promoter regions of the 56 *SiPUB*s. The count of *cis*-acting elements exhibited variability, ranging from 95 (*SiPUB24*) to 235 (*SiPUB37*), with an average of 157. These elements can be categorized into seven distinct functional groups: hormone-responsive elements, elements associated with plant growth and development, stress-responsive elements, light-responsive elements, site-binding elements, promoter-related elements, and other functional elements. The results revealed that most *SiPUB* promoters contained a CAAT or TATA box, which were commonly found *cis*-acting elements in the promoter and enhancer regions. The specific elements associated with light response, hormone response, promoter-related functions, and developmental processes widely spread among *SiPUB*s, suggesting a potential role for *SiPUB*s in various biological activities. Moreover, we identified a diverse set of 16 *cis*-acting elements associated with environmental stress responses. For instance, 53 *SiPUBs* contained the MYB element, which is involved in environmental adaptation and secondary metabolic regulation. Forty-six *SiPUBs* harbored the AAGAA-motif of the ABA elements, while 54 contained the ABA-responsive element (ARE) and as-1, which are associated with various stresses, including hypoxia, salicylic acid, cold, drought, and pathogen attacks. Notably, we detected that all 56 *SiPUB* genes contained 15 intriguing *cis*-acting elements predominantly associated with drought and other environmental stresses (**Supplementary Table S6**; **Figure 2D**). These results indicate that *SiPUB* genes are specifically involved in functions related to environmental stresses.

### Expression analysis of *SiPUB* genes under drought stress

**Figure 4:**
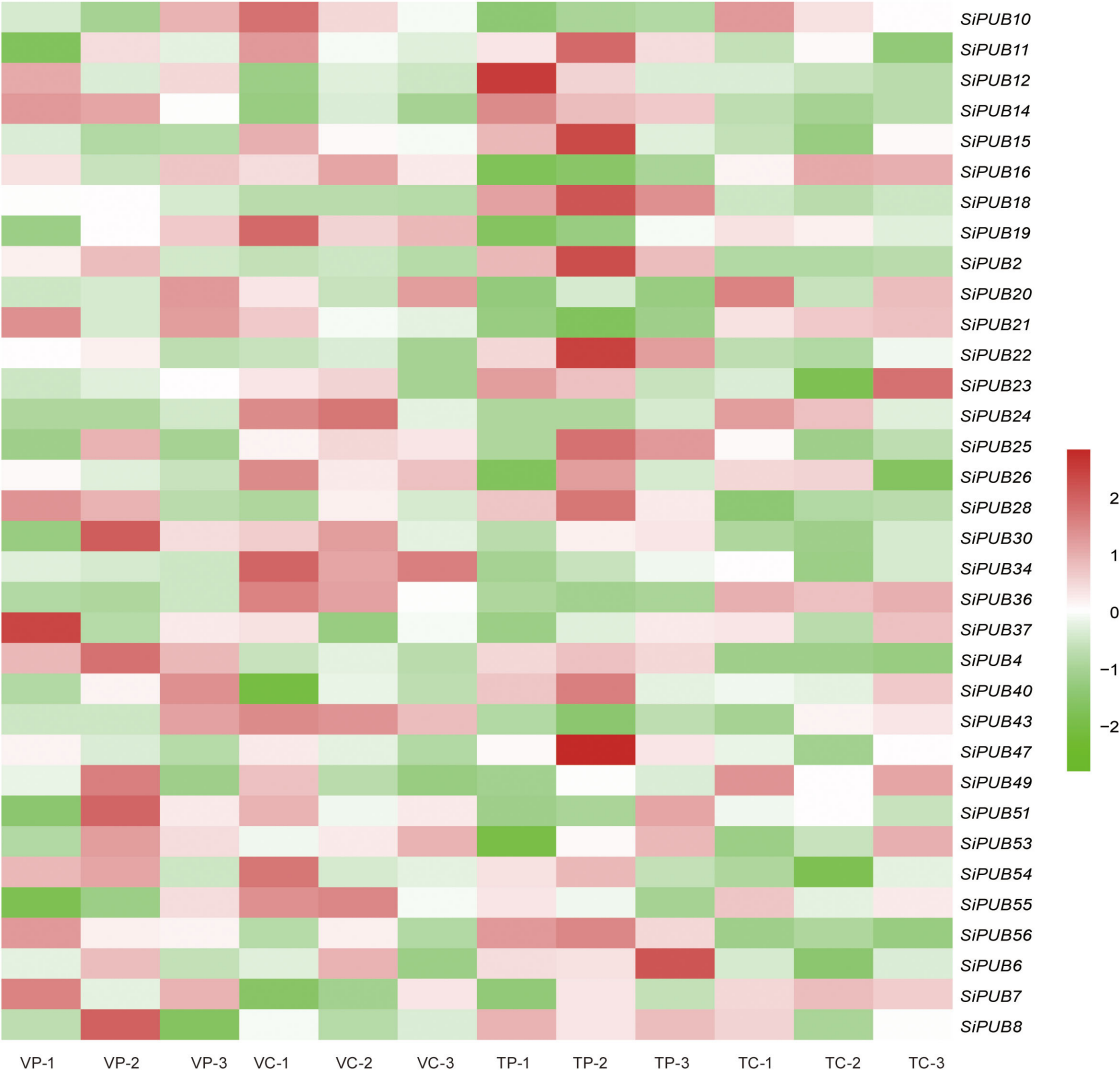
Expression analysis of the *SiPUB* genes under drought stress. Heatmaps are generated using 4 groups of public RNA-seq data. VP and TP indicate the treatments of drought-tolerant genotype TEX-1 (T) and the drought-sensitive genotype VEN-1 (V) under drought stress. VC and TC indicate the controls of drought-tolerant genotype TEX-1 (T) and the drought-sensitive genotype VEN-1 (V) under normal environment. The color code represents the log2-transformed FPKM value. High and low expression levels are shown in dark red and bright green, respectively.

To further explore the function of *SiPUB* genes in response to drought stress, we analyzed the expression profiles of all 56 *SiPUB*s based on the four groups of RNA-Seq transcriptome data on sesame available on the NCBI database. Thirty-four (60.7%) *SiPUB*s displayed differential expression under drought stress. Next, we analyzed the expression heatmaps of the 34 *SiPUB* genes with the log2FC values (**Figure 4**). The differential multiple matrices of these *SiPUBs* are displayed in **Supplementary Table S7**. Nine genes were differentially expressed with a |log2FC| > 1 under four drought treatments (**Supplementary Figure S2**). The results suggested that the *SiPUB* genes are widely involved in drought tolerance in sesame.

### Different expression of *SiPUB* genes in various tissues

**Figure 5:**
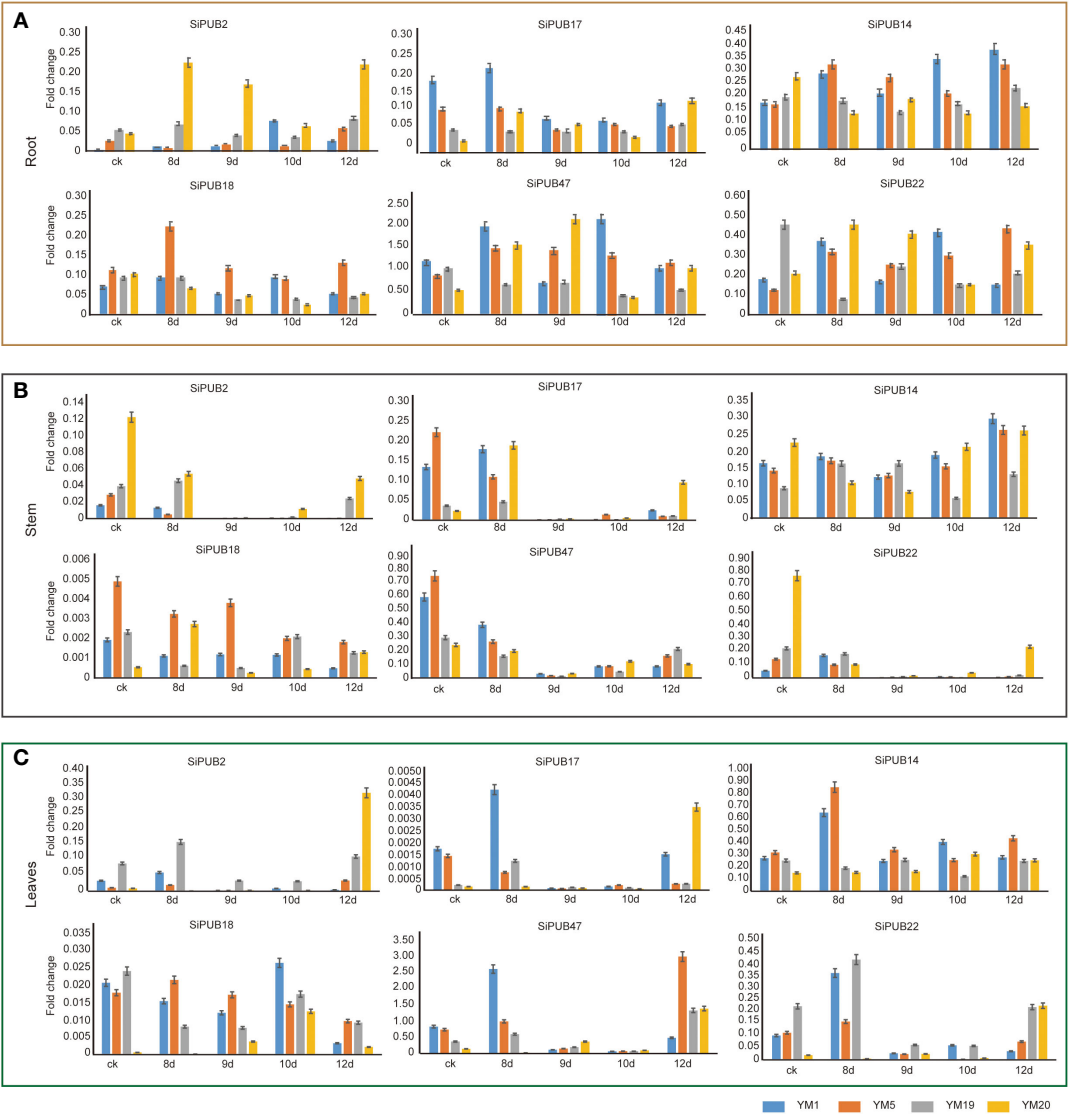
Validation of 6 *SiPUB* genes by qRT-PCR in different tissues of four sesame accessions under drought treatment. The root **(A)**, stem **(B)**, and leaves **(C)** of two drought- tolerant sesame accessions (YM19 and YM20) and two drought-sensitive accessions (YM1 and YM5) are treated under 8d, 9d, 10d, and 12d for qRT-PCR validation. X-axis represents different treat time. Y-axis represents the relative expression level of each *PUB* gene. YM1, YM5, YM19, and YM20 are shown in blue, orange, grey, and yellow column, respectively.

To validate the reliability of the transcriptome sequence analysis, we selected 6 *SiPUB* genes (*SiPUB2*, *SiPUB14*, *SiPUB17*, *SiPUB18*, *SiPUB22*, and *SiPUB47*) based on their distinct expression patterns observed in the transcriptome data and performed qRT-PCR validation.The expression levels of these six genes were assayed across three different tissues in two drought- tolerant (YM19 and YM20) and two drought-sensitive sesame accessions (YM1 and YM5) (**Figure 5**). All six *SiPUB* genes displayed diverse expression patterns across the various sesame tissues. In the root tissue (**Figure 5A**), the expression level of all 6 *SiPUB* genes increased during the 8-day drought exposure in drought-tolerant and drought-sensitive varieties. Among those, *SiPUB47* exhibited a notably higher expression level and surpassed the other five *SiPUB* genes by a significant margin (four to eight times). In the stem tissue (**Figure 5B**), the expression level of *SiPUB2* and *SiPUB22* was lower in the drought-sensitive varieties than in the drought-tolerant plants. The two genes showed a general down-regulation trend. Conversely, *SiPUB14* exhibited a consistent upregulation in response to drought stress across all samples. *SiPUB18* displayed the significantly lowest expression level compared to the other five *SiPUB* genes. In leaf tissues (**Figure 5C**), *SiPUB47* and *SiPUB14* displayed the significantly highest expression level compared to the other four *SiPUBs*. On the other hand, *SiPUB17* exhibited the lowest expression, with levels more than 10 times lower than those of the other *SiPUB* genes. The qRT-PCR results of the 6 *SiPUB* genes indicated different expression profiles and matched the expression patterns observed in the abovementioned transcriptomic analyses. The results suggest that the *SiPUB* gene family potentially functions in different tissues and contributes to drought tolerance capabilities in different cultivated sesame varieties.

### 3D structure and protein-protein interaction analysis of SiPUBs

**Figure 6:**
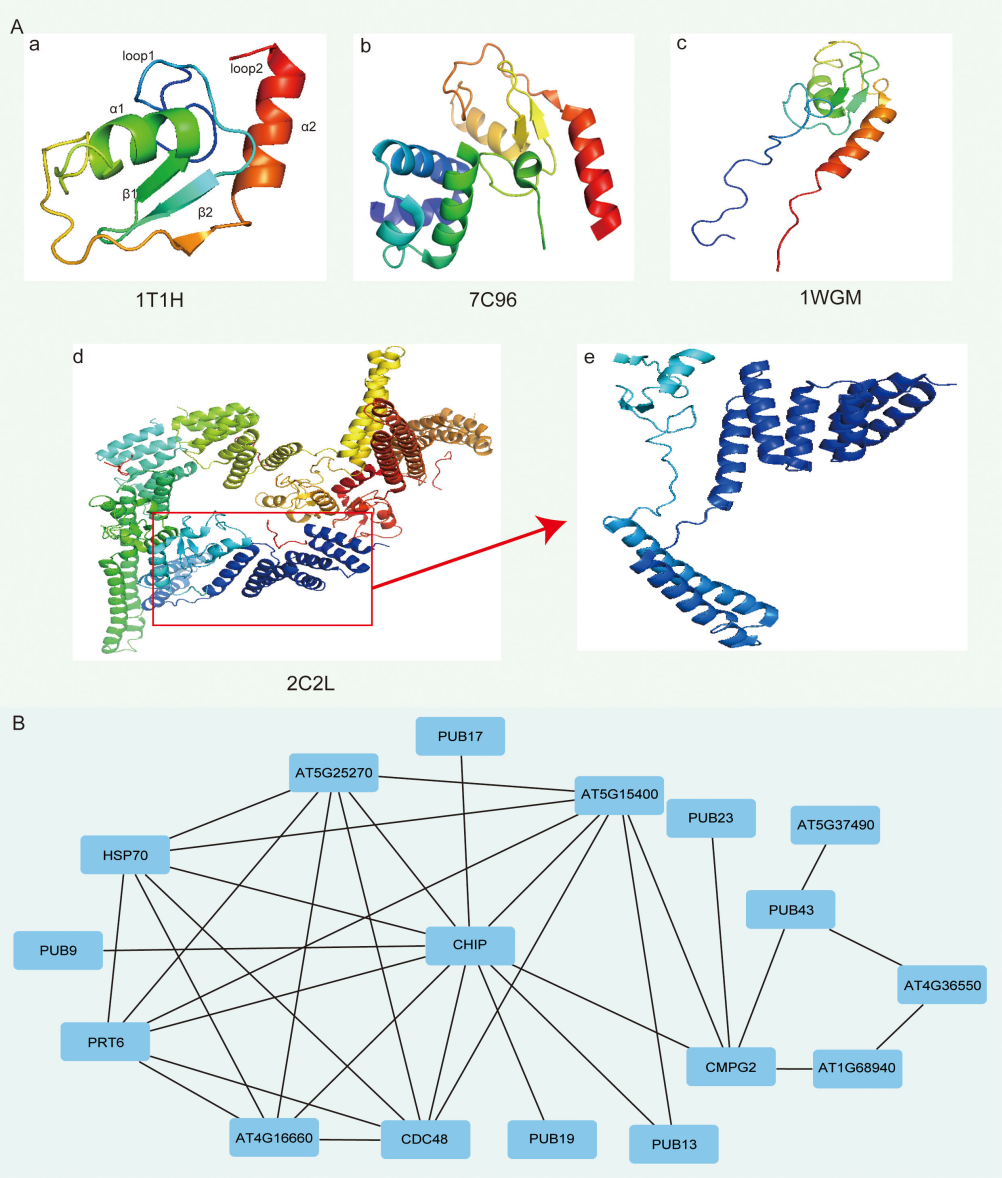
Three-dimensional (3D) structure and protein-protein interaction (PPI) networks of SiPUB proteins. **(A)** Four structure models of SiPUBs. 1T1H (a), 7C96 (b), 1WGM (c), and 2C2L (d) are determined using RCSC PDB. **(B)** PPI analysis of SiPUBs with homologous proteins in *Arabidopsis* (AtPUBs). The homologous annotation of each protein between SiPUB and AtPUB is conducted using the STRING database.

To further differentiate the higher structural characteristics of SiPUB proteins, we performed an RCSB PDB analysis and successfully determined a set of four distinct structural types for the 56 SiPUB proteins (**Figure 6A**). A significant majority of SiPUB proteins (95%, 53 out of 56) were categorized under the PDB IDs of 1T1H and 7C96. SiPUB12 and SiPUB14 showed a 2C2L structure, while SiPUB19 was classified under the 1WGM structure (**Supplementary Table S8**). All of these four structures exhibited common structures with a central α-helix (α1), a C-terminal helix (α2), a small antiparallel β-sheet (β1 and β2), and two distinct loops (loop1 and loop2) (**Figure 6A-a**).

To explore a potential interaction between the proteins, we conducted an interaction network analysis of the SiPUB proteins by comparing them with their homologs in *A. thaliana* (AtPUBs). Of the 60 AtPUBs examined, 34 (57%) exhibited homology with the 56 SiPUBs (**Supplementary Table S9**). Based on the STRING database, the PUB proteins displayed extensive connections and interactions with 11 specific proteins, such as HSP70, CMPG2, and other stressed-related proteins (**Figure 6B**). The intricate protein networks of protein-protein interactions enhance our understanding of molecular mechanisms and biological processes involving *SiPUB*s.

## Discussion

The *PUB* gene family plays a significant role in response to various abiotic stresses and has been identified in numerous plant species. Extensive research suggests that *PUB* genes participate in drought tolerance in many plants, such rice, *Arabidopsis*, and wheat (Qin et al., 2020; Seo et al., 2021). Sesame is an ancient oilseed crop with high tolerance to drought and high temperature. Identifying the function and expression patterns of *PUB* genes in response to drought will supply a key aid for genetics and breeding research in sesame and other crops. In this study, we screened *SiPUB* genes from the sesame reference genome (var. Yuzhi 11) and validated the potential function of *SiPUB* genes in drought stress responses in drought-tolerant and drought-susceptible accessions for the first time.

### Phylogenetic structure, gene structure, and *cis*-acting elements of *SiPUB*s

We screened all sesame *PUB* genes based on combined results of HMM and BLAST searches. Fifty-eight candidate *SiPUB* genes were preliminarily identified in the whole genome. We used the PfamScan tool (v1.6) to verify the accuracy of the candidate *PUB* genes. After the filtering process, two candidate genes were excluded from consideration due to the absence of a U-box domain or repeat sequences. As a result, a total of 56 non-redundant *SiPUB* genes were ultimately obtained. All the *SiPUB* genes displayed a complete U-box domains. However, the number of *PUB* genes in sesame (56) is relatively lower than in rice (76) (Zeng et al., 2008), soybean (125) (Wang et al., 2015), barley (67) (Ryu et al., 2019), cotton (93~208) (Lu et al., 2020), and banana (91) (Hu et al., 2018). The variation in the genome size with evolution might attribute to the variation in the number of *PUB* genes in different species.

In sesame, 56 *SiPUBs* exhibited an uneven distribution across the 13 chromosomes and could be categorized into six groups based on multi-alignments and phylogenetic analysis (**Figures 2A**, **3A**). Phylogenetic analysis including other plant species indicated that 192 PUB proteins from three species (56 from sesame, 76 from rice, and 60 from *Arabidopsis*) were classified into five groups (I-V) (**Figure 1**). All five groups displayed substantial similarities due to the common presence of the core U-box domain. The grouping pattern in the phylogenetic tree exhibited a high resemblance to other species, such as Chinese cabbage, cotton, and banana (Wang et al., 2015; Hu et al., 2018; Lu et al., 2020). Notably, sesame and *Arabidopsis PUB* genes were clustered together in a subclade. Additionally, we observed 45 orthologous gene pairs between *SiPUB*s and *AtPUB*s, surpassing the number between *SiPUB*s and *OsPUB*s (23) (**Figure 3C**). Our findings suggest a closer evolutionary relationship between sesame and *Arabidopsis* than with rice. *SiPUB*s might possess similar functions to their orthologous counterparts in *Arabidopsis* (Cho et al., 2008; Chen et al., 2021).

Notably, *SiPUB* genes within the same group displayed similar gene structures, whereas structural variations were observed among different groups (**Figures 2B, C**). *SiPUB*s possess a conserved U-box domain, which is typically associated with ARM, anaphase-promoting complex subunit 3 (ANAPC3), and WD40 repeats (**Figure 2B**). Notably, many biologically functional PUB proteins have been characterized as U-box proteins with ARM repeats (Wang et al., 2020; Wang et al., 2021). Among the identified *SiPUB* genes, 24 genes were found to exclusively contain the U-box domain, while 22 genes possessed the U-box domain and ARM repeats (**Supplementary Table S4**). ARM repeats primarily mediate interaction with substrates, indicating that ubiquitination occurs in response to interactions (Shu and Yang, 2017; Al-Saharin et al., 2022). Based on the diverse gene structures identified, we inferred that *SiPUB* genes in sesame likely have various biological functions.

In addition, diverse domains were present among the *SiPUB* genes within the same group (**Figure 2B**). Group 1 comprises ten genes, most of which exhibit the presence of the U-box domain and other domains, such as ANAPC3 repeats, protein tyrosine and serine/threonine kinase (PK_Tyr_Ser-Thr), protein kinase (Pkinase), and ubiquitin elongating factor core (Ufd2P_core) family. ANAPC3 belongs to the anaphase-promoting complex subunits, a multiprotein subunit E3 ubiquitin ligase complex that controls chromosome segregation and mitotic exit in eukaryotes (Peters, 2002; Vodermaier et al., 2003; Peters, 2006). The PK_Tyr_Ser-Thr and Pkinase domains are part of the receptor-like kinase (RLK) gene families, which have essential roles in resistance signaling pathways against various abiotic stresses. Previous studies have suggested that these domains could have stress-responsive functions under drought or *Fusarium graminearum* treatments in wheat (Yan et al., 2023). *SiPUB52*, which possesses a WD40 repeat in its structure, was found to be distinct from other *SiPUB*s and did not cluster into any of the identified groups in the species tree (**Figure 2A**). A *LIN* gene containing U-box and WD40 repeat domains is central in the nodulation process of rhizobia and controls the early infection of rhizobia in leguminous plants (Kiss et al., 2009). Similar observations have been reported in other crops, suggesting that the diverse functions of *PUB*s may be associated with different motifs (Lu et al., 2020; Wang et al., 2021). Therefore, *SiPUB52* might also conduct a similar function in sesame and potentially contribute to disease resistance.

Regulatory elements for a gene are typically found in its upstream region, with their functional impact generally extending within a range of 2,000 base pairs (Doane and Elemento, 2017). *Cis*-acting elements within gene promoter regions play a vital role in the regulation of gene expression across various crops, particularly in response to multiple abiotic stresses and plant growth (Zhang et al., 2020; Riaz et al., 2021; Rehman et al., 2022; Cui et al., 2023). The *cis*-acting elements of the putative promoters of *PUB* genes are involved in many key biological processes, including stress responses, hormonal regulation, and crop growth and development (Wang et al., 2021; Chen et al., 2022). In our study, all 56 *SiPUB* genes contained stress-related elements in their putative promoter regions (**Figure 2D**). The number of stress-related element types in these *SiPUB* genes varied from 3 (*SiPUB15*) to 11 (*SiPUB16*, *SiPUB18*, and *SiPUB25*), suggesting a role for *PUB* genes in stress tolerance in sesame. Among these *cis*-acting elements, 18 involved in environmental stress responses, such as ARE, dehydration-responsive element (DRE), and MYB, were included (**Figure 2D**). ARE is a crucial regulator of plant drought stress response and could enhance crops’ drought tolerance by regulating the ARE-dependent ABA signaling (Fujita et al., 2005; Roca Paixão et al., 2019). DRE is an important transcription factor in regulating responses to abiotic stress in many plants, such as *Brassica napus* and rice (Zhao et al., 2006; Khan et al., 2017; Huang et al., 2018). The transgenic expression of *LcDREB3a* from *Leymus chinensis* has been reported to enhance drought and salt tolerance in *Arabidopsis* (Xianjun et al., 2011). DREB2 has also been proven to improve the drought tolerance of *Broussonetia papyrifera* (Sun et al., 2014). The MYB family of transcription factors are widely involved in many plants, such as *Pyrus betulaefolia*, barley, and wheat, in their adaptive responses to drought stress (Bi et al., 2016; Li et al., 2017; Alexander et al., 2019). Further evidence indicated that MYBs act as mediators of ABA action in response to drought stress and could be a valuable target for stress tolerance engineering in crop improvement (Baldoni et al., 2015; Xu et al., 2019).

Our study detected that most *SiPUB* genes (53 out of 56) contained MYB elements. Moreover, several other stress-responsive *cis*-regulatory elements, including W-box, MBS, TC-rich repeats, as-1 elements, and WUN-motif, were also present in the *SiPUB* genes. These findings imply that *SiPUB* genes could play a crucial role in various mechanisms related to abiotic stress tolerance. Therefore, they can offer promising prospects for future breeding endeavors on improving abiotic stress tolerance in sesame.

### Functional potential of differentially expressed *SiPUBs*

Based on public RNA-seq datasets, we identified nine *SiPUB* genes differentially regulated under drought stress conditions and exhibiting a potential function in response to drought (**Figures 4**, **S2**). In rice, *OsPUB67* is positively correlated with drought tolerance (Qin et al., 2020). We found that *SiPUB28* and *SiPUB43* were grouped with 11 *OsPUB* and 2 *AtPUB* genes (Group I in **Figure 1**) and presented a similar potential to *OsPUB67* in drought tolerance. Similarly, in barley, several *HvPUB*s have been identified as negative or positive regulators of drought response (Ryu et al., 2019). These findings reflect the similar functions of *PUB* genes in drought stress responses across various plant species.

In *Phyllostachys edulis*, *PePUB60* and *PePUB120* are upregulated under drought stress conditions (Zhou et al., 2021). In *Arabidopsis*, *AtPUB46* is a positive regulator of drought response, as overexpression of this gene resulted in increased tolerance, while knockout mutants exhibited hypersensitivity to drought (Adler et al., 2018). In this article, we observed that three *SiPUB* genes (*SiPUB11*, *SiPUB15*, and *SiPUB50*) clustered into one branch with four *AtPUB*s (*AtPUB9*, *AtPUB46*, *AtPUB47*, and *AtPUB49*) and displayed roles possibly similar to *AtPUB46* in drought tolerance. These studies suggest a central role for *SiPUB* genes in drought stress adaptation in sesame (Wang et al., 2021; Chen et al., 2022).

To explore the functions of *PUB* genes in sesame, we investigated the expression levels of six *SiPUB* genes in three different tissues under different drought treatments using qRT-PCR (**Figure 5**). The results indicated that five *SiPUB* genes (*SiPUB2*, *SiPUB17*, *SiPUB18*, *SiPUB47*, and *SiPUB22*) exhibited higher expression levels in sesame roots (**Figure 5A**) than in the stem (**Figure 5B**) and leaf tissues (**Figure 5C**). In *Arabidopsis*, mutations in *AtPUB22* and *AtPUB23* enhanced drought tolerance compared to wild-type plants (Cho et al., 2008). In our analysis, *SiPUB17*, *SiPUB42*, and *SiPUB47*, which were clustered together with *AtPUB22* and *AtPUB23* in Group II (**Figure 1**), exhibited more than 50% homology at the amino acid level to *AtPUB22* and *AtPUB23*. *AtPUB17* is a positive regulator of plant defense and stress signaling responses, whereas the closely related rice orthologues *OsPUB2* and *OsPUB3* act as positive regulators, specifically in temperature stress responses (Yang et al., 2006; Byun et al., 2017).

In addition, based on our phylogenetic analysis, two *SiPUB* genes (*SiPUB46* and *SiPUB55*) were clustered together with the previously mentioned three genes in group V (**Figure 1**). Interestingly, we observed that *SiPUB55* was downregulated in the drought-sensitive variety VEN-1 under drought stress, but was upregulated in the drought-tolerant variety TEX-1 (**Figure 4**). We infer that *SiPUB55* might potentially serve as a negative regulator in response to drought stress in sesame. Moreover, *SiPUB14* displayed the highest expression in leaves, more than twice times as high as in the root and stem tissues (**Figure 5**). This strong concordance between the qRT-PCR results and transcriptome analyses provides compelling evidence for the crucial role of *SiPUB* genes in drought stress regulation in sesame.

In this article, we analyzed the 3D structure of PUBs to comprehend the sequence patterns, functions, binding sites, and interactions of candidate proteins with other targets. These models provide valuable insights into the structural characteristics and potential functions of the SiPUB proteins in sesame. According to the RCSC PDB database, four comparative homology models for 56 SiPUB proteins were available. The significant sequence identity ranged from 31% to 93% (**Figure 6A**; **Supplementary Table S8**). All four models shared common structural features of PUBs, including two helices (α1 and α2), two small antiparallel β-sheets (β1 and β2), and two distinct loops (loop1 and loop2) (**Figure 6A**). Most SiPUB proteins (95%) were associated with PDB IDs 1T1H and 7C96. These structures are also present in AtPUB14 and GmPUB13 (Andersen et al., 2004; Lin Y. et al., 2021). Moreover, two PUB proteins (SiPUB12 and SiPUB14) corresponded to the part residues of 2C2L, while only SiPUB19 matched the PDB ID 1WGM (**Figure 6A(c)**). The structural similarity among these proteins suggests the conserved overall fold and potential function. The screened network of SiPUB putative interacting proteins in sesame and *Arabidopsis* on the STRING database indicated a high conservation of PUB proteins (**Figure 6B**). As the confidence score of these functional proteins was set at 0.4, the connections between the nodes and lines had a high degree of credibility. For the extensively connected network between PUBs and specific proteins, AtCHIP (homologs of *SiPUB12*/*14*) interacted with a key component and connected with 11 other proteins. *AtCHIP* positively regulates the homeostasis of caseinolytic peptidase (Clp) proteolytic subunits, thereby maximizing the production of functional chloroplasts (Wei et al., 2015). In addition, AT5G25270, PRT6, and CDC48 are all ubiquitin-related proteins, indicating that *PUB* genes have broad connections with other ubiquitin genes and are central in the ubiquitination process. In the future, we shall perform extensive analysis of the above key *SiPUB* genes involved in the interacting protein network and decipher the regulation patterns of *PUB* genes in response to drought stress in sesame and other crops.

## Conclusion

We identified 56 *SiPUB* members in the sesame genome and comprehensively analyzed their distribution, phylogenetic evolution, structure, and expression profiles. All 56 *SiPUB* genes were classified into six distinct groups. U-box motifs and 18 key *cis*-acting elements were detected in putative promoters of *SiPUB* genes. Expression profile comparison analysis of *SiPUB* genes using transcriptome data and qRT-PCR in drought-tolerant and drought-susceptible accessions validated the potential function of those genes in response to drought. These findings contribute to a comprehensive understanding of the regulatory mechanism of *PUB* genes in drought stress in sesame.

## Data availability statement

The datasets presented in this study can be found in online repositories. The names of the repository/repositories and accession number(s) can be found in the article/**Supplementary Material**.

## Ethics statement

The sesame varieties Henan No. 1, Yuzhi No. 3, Zhengheizhi No.1, and Zhengheizhi No.4 used in this study were planted and grown in Yuanyang, Henan Province, China. All the studies are not involved in ethics problems.

## Author contributions

HC: Data curation, Software, Writing – original draft, Funding acquisition, Investigation, Methodology, Visualization. QT: Data curation, Validation, Writing – review & editing. MJ: Data curation, Investigation, Writing – review & editing. YD: Investigation, Writing – review & editing. GL: Data curation, Writing – review & editing. QM: Investigation, Writing – review & editing. HZ: Conceptualization, Funding acquisition, Methodology, Project administration, Supervision, Writing – review & editing. XZ: Conceptualization, Funding acquisition, Project administration, Supervision, Writing – review & editing. HM: Conceptualization, Funding acquisition, Project administration, Resources, Writing – original draft, Writing – review & editing.

## Supplementary material

Supplementary Figure S1 Multiple alignments of U-box domains of SiPUB proteins. The protein sequences were aligned using MAFFT, and the resulting alignments were visualized and displayed using Overleaf. The U-box domains were highlighted with a red frame to facilitate their identification.

Supplementary Figure S2 A venn diagram of the relationship between differentially expressed gene (DEG) groups. The numbers in each section of the Venn diagram indicated the number of DEGs in each respective DEG group. The Venn diagram provided a visual representation of the overlapping and unique DEGs among the different DEG groups.

Supplementary Table S1 Primers for qRT-PCR analysis of selected PUB genes in sesame.

Supplementary Table S2 Summary of the detailed characterization of 56 PUB genes in sesame.

Supplementary Table S3 A list of AtPUBs and OsPUBs used in phylogenetic analysis with SiPUBs.

Supplementary Table S4 The Pfam analysis of SiPUB proteins in this study.

Supplementary Table S5 The tandem duplication block types of 56 SiPUB genes.

Supplementary Table S6 Cis-Acting elements analysis in the 2 kb promoters of SiPUB genes.

Supplementary Table S7 Transcriptomic data for the expression analysis of drought-tolerant genotype TEX-1(T) and drought-sensitive genotype VEN-1(V).

Supplementary Table S8 The three-dimensional (3D) structure analysis of 56 SiPUB proteins.

Supplementary Table S9 The homologous relationships between AtPUB and SiPUB genes.
