# Supplementary figures and images for: Genome-wide analysis of the U-box E3 ubiquitin ligase family role in drought tolerance in sesame (Sesamum indicum L.)
Source: Front Plant Sci. 2023 Sep 19;14:1261238. doi: 10.3389/fpls.2023.1261238 (PMC10558006; doi:10.3389/fpls.2023.1261238)

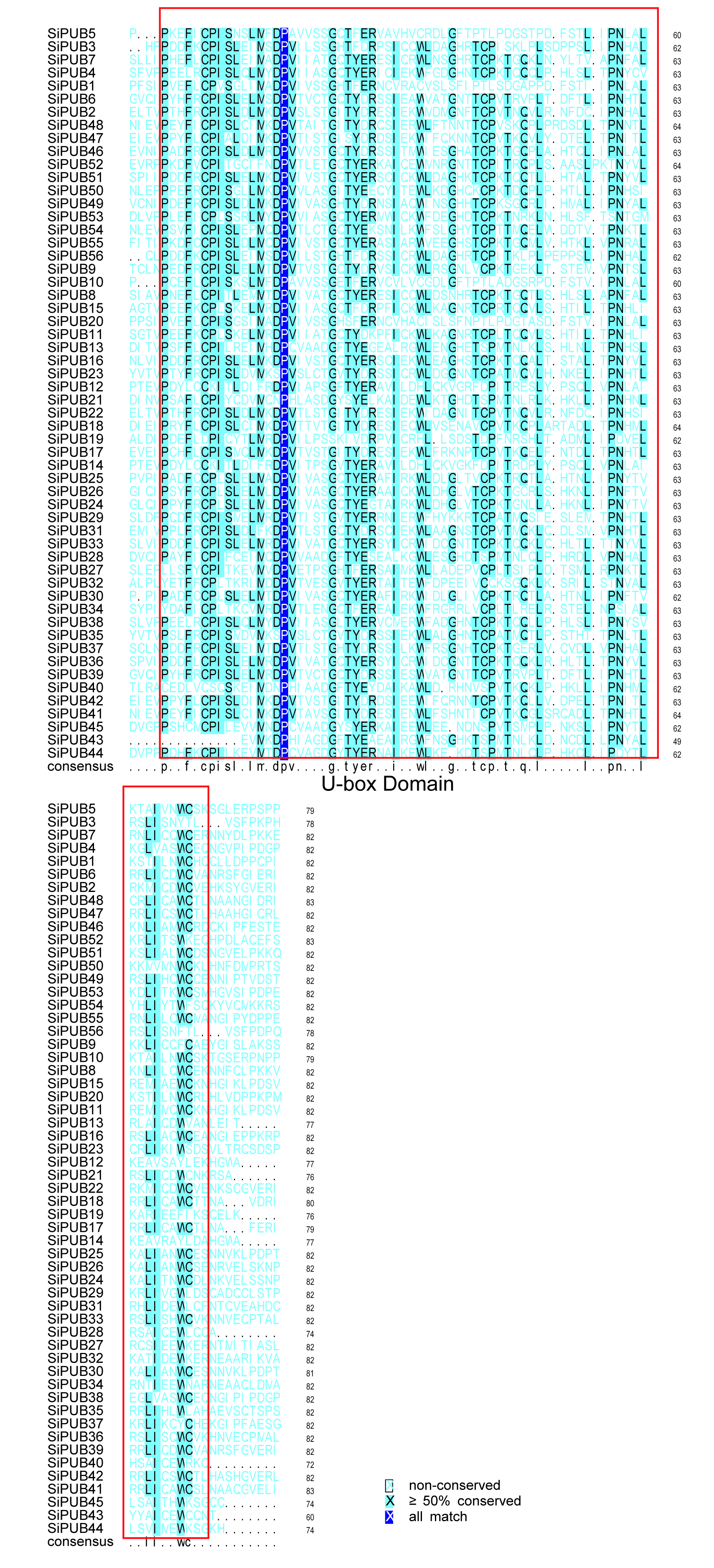

Supplement: Supplementary Figure S1 — Multiple alignments of U-box domains of SiPUB proteins. The protein sequences were aligned using MAFFT, and the resulting alignments were visualized and displayed using Overleaf. The U-box domains were highlighted with a red frame to facilitate their identification. [file Image_1.tif]

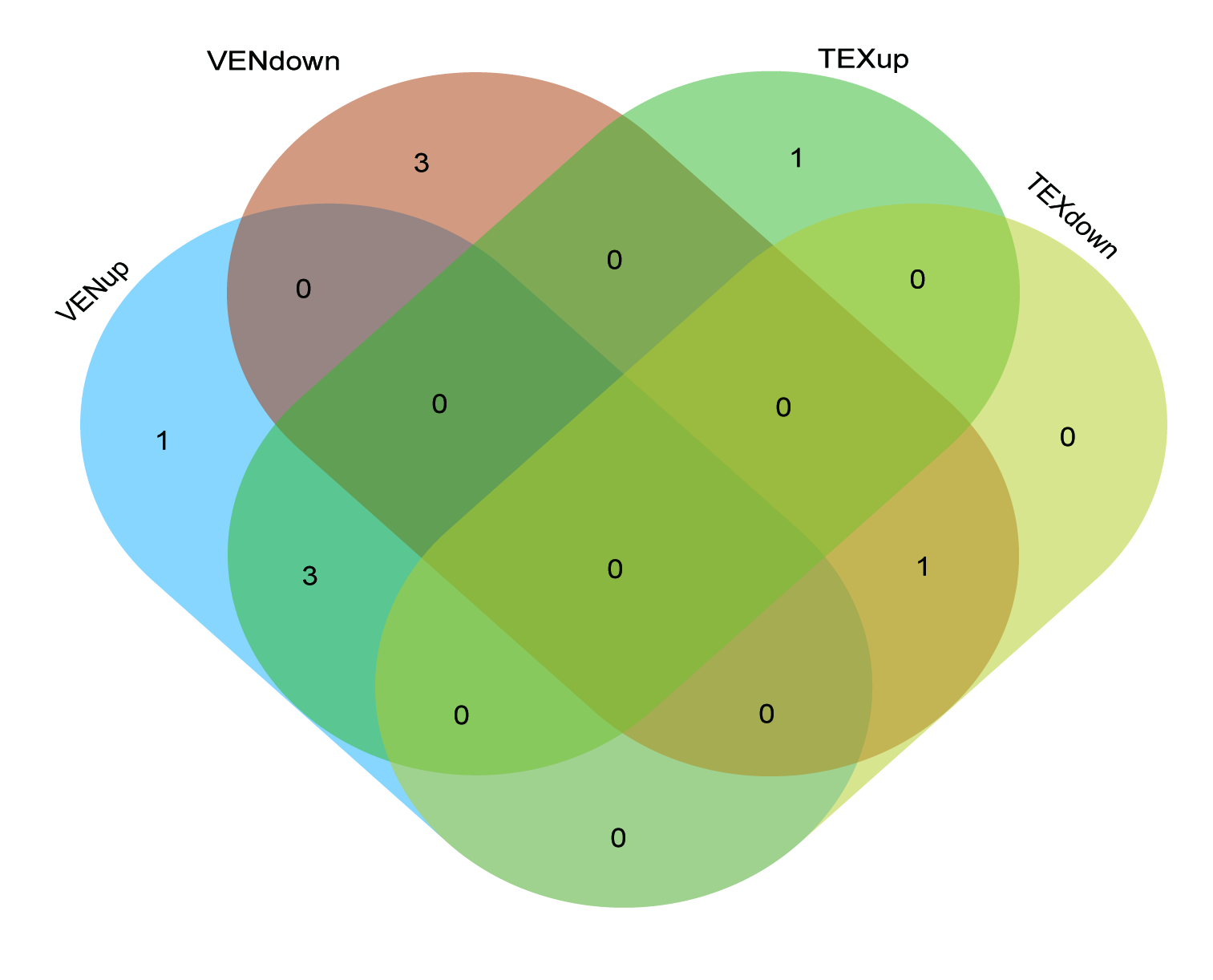

Supplement: Supplementary Figure S2 — A venn diagram of the relationship between differentially expressed gene (DEG) groups. The numbers in each section of the Venn diagram indicated the number of DEGs in each respective DEG group. The Venn diagram provided a visual representation of the overlapping and unique DEGs among the different DEG groups. [file Image_2.tif]
