# Supplementary material for: Genome-wide analysis of the U-box E3 ubiquitin ligase family role in drought tolerance in sesame (Sesamum indicum L.)
Source: Front Plant Sci. 2023 Sep 19;14:1261238. doi: 10.3389/fpls.2023.1261238 (PMC10558006; doi:10.3389/fpls.2023.1261238)
Supplement: Supplementary Table S1 — Primers for qRT-PCR analysis of selected PUB genes in sesame. [file Table_1.docx]

| Gene ID | Primers(5’ -- 3’) |
| --- | --- |
| SiPUB2 | Forward: CGACAGCATTCTCTATCTCA |
|  | Reverse: CTTGTAGCACCACCAGTAG |
| SiPUB17 | Forward: CGGTGAGGATTCTGAGTT |
|  | Reverse: GGAACACAAGCACTATCTC |
| SiPUB14 | Forward: CGCCATTGATGCTTATACTG |
|  | Reverse: AATCCTCCTCCACTCTTGT |
| SiPUB18 | Forward: GACCAAGAACCACCAGTT |
|  | Reverse: CTTAACGCTTCCTCAAGAC |
| SiPUB47 | Forward: CGCTAACGGTGTTGGATT |
|  | Reverse: CAGGAGTTGCCGAGAATT |
| SiPUB22 | Forward: GCTCACAGTTCCTACACAT |
|  | Reverse: CGGCATCTATCCACTTCTC |
| SiTub | Forward: TGGTGACCTCAACCACCTCAT |
|  | Reverse: TGACAGCGAGTTTCCTGAGATC |
